# Use of the *Saccharomycopsis schoenii MET17* promoter for regulated heterologous gene expression

**DOI:** 10.1007/s00294-024-01294-6

**Published:** 2024-07-01

**Authors:** Mareike Rij, Jürgen Wendland

**Affiliations:** 1 Department of Microbiology and Biochemistry, Hochschule Geisenheim University, Von-Lade-Straße 1, 65366 Geisenheim, Germany; 2 Geisenheim Yeast Breeding Center, Hochschule Geisenheim University, Von-Lade-Straße 1, 65366 Geisenheim, Germany

**Keywords:** Predator yeast, Tool box, Inducible/repressible promoter, Methionine biosynthesis, RNAseq, Reporter gene

## Abstract

The ability to regulate the expression of genes is a central tool for the characterization of fungal genes. This is of particular interest to study genes required for specific processes or the effect of genes expressed only under specific conditions. *Saccharomycopsis* species show a unique property of necrotrophic mycoparasitism that is activated upon starvation. Here we describe the use of the *MET17* promoter of *S. schoenii* as a tool to regulate gene expression based on the availability of methionine. Conditional expression was tested using *lacZ* and *GFP* reporter genes. Gene expression could be strongly down-regulated by the addition of methionine or cysteine to the growth medium and upregulated by starvation for methionine. We used X-gal (5-bromo-4-chloro-3-indolyl-β-d-galactopyranoside) to detect *lacZ*-expression in plate assays and ONPG (ortho-nitrophenyl-β-galactopyranoside) as a substrate for β-galactosidase in liquid-phase assays. For in vivo expression analyses we used fluorescence microscopy for the detection and localization of a *MET17*-driven histone *H4*-*GFP* reporter gene. With these assays we demonstrated the usefulness of the *MET17* promoter to regulate expression of genes based on methionine availability. *In silico* analyses revealed similar promoter motifs as found in *MET3* genes of *Saccharomyces cerevisiae* and *Ashbya gossypii*. This suggests a regulation of the *MET17 *promoter by *CBF1* and *MET31/MET32* in conjunction with the transcriptional activator *MET4*, which were also identified in the *S. schoenii* genome.

## Abstract (plain language summary)

This article describes the characterization of the *S. schoenii MET17* promoter for regulated gene expression.

## Introduction

Growing interest in the biology of *Saccharomycopsis* species aims at the elucidation of its necrotrophic mycoparasitism. *S. schoenii* and other predator yeasts of this genus target prey cells by penetration pegs that lead to killing of the prey and uptake of prey nutrients by the predator (Lachance and Pang 1997; Junker et al. 2019). Recent comparative genomics studies indicated that *Saccharomycopsis* species lack several genes for the uptake and assimilation of sulphate (Junker et al. 2017, 2019; Hesselbart et al. 2018). Sulphate uptake mutants are resistant to otherwise toxic levels of selenate, which provides a convenient selection method for the isolation of *Saccharomycopsis* species (Lachance and Pang 1997; Dost et al. 2024). Starvation for sulphur compounds can easily be triggered by lack of methionine in growth media and starvation is a prerequisite for predacious activity.

*Saccharomycopsis* utilize an alternative codon usage as they translate CUG as serine instead of leucine (Krassowski et al. 2018; Muhlhausen et al. 2018; Junker et al. 2019). To initiate molecular genetic studies we development a tool-box for gene-function analyses in *Saccharomycopsis* (Kayacan et al. 2019; Kaimenyi et al. 2023). This tool-box contains a transformation protocol, several marker genes as well as several constitutively active promoters. However, a promoter for a regulated expression of target genes was not available.

In other systems regulatable promoters are derived from various sources. In *Saccharomyces cerevisiae* galactose-inducible promoters derived from the *GAL1*, *GAL7* or *GAL10* genes, have been used for the strong expression of genes (Brunelli and Pall 1993; Sil et al. 2000). Another sugar-inducible promoter available in *S. cerevisiae* is the *MAL32* promoter that is activated by maltose and repressed by glucose (Meurer et al. 2017). Sugar-regulated promoters have been utilized in other systems as well, e.g. xylose-dependent gene expression in *Chaetomium thermophilum* (Reislöhner et al., 2023). Recombinant protein expression in *Komagataella phaffii* (formerly *Pichia pastoris*) relies on the methanol-inducibility of the *AOX1* promoter, while in *Aspergillus nidulans* the *alcA* promoter is used for alcohol-induced gene expression (Waring et al. 1989; Raschke et al. 1996). Other modes of regulation employ the thiamine (vitamin B_1_)-repressible *nmt1* promoter in *Schizosaccharomyces pombe*, the light-inducible *vvd* promoter in *Neurospora crassa* or doxycycline on/off-regulatable *tet*-promoters in *S. cerevisiae* and other fungi (Moreno et al. 2000; Hurley et al. 2012; Kluge et al. 2018). Feedback-inhibition of the methionine biosynthesis pathway via methionine allows regulatable gene expression by using *MET3* or *MET17* promoters, e.g. in *Ashbya gossypii*, *Candida albicans* or *S. cerevisiae* (Cherest et al. 1985; Thomas et al., 1989; Care et al. 1999; Dünkler and Wendland, 2007).

In this paper, we describe our efforts to establish a regulatable gene expression system in the predator yeast *S. schoenii*. Due to lack of homologs of the widely used *GAL*-, *MAL*- or *MET3*-promoters we targeted the *S. schoenii MET17* promoter and employed *lacZ* and *GFP* as heterologous reporter genes. Regulatable expression via the *SsMET17*-promoter could be achieved via the addition or removal of methionine in the growth media. This adds a further tool to the functional analysis repertoire for *Saccharomycopsis* species.

## Materials and methods

### Strains and culture conditions

The *Saccharomyces cerevisiae* strain BY4741 (*MATa; his3Δ1; leu2Δ0; met1Δ0; ura3Δ0*; Euroscarf, Oberursel, Germany) and the *Saccharomycopsis schoenii* strain CBS 7425 (wild type, Westerdijk Institute, Utrecht, The Netherlands) and its derivatives were propagated in YPD (1% yeast extract, 2% casein peptone and 2% dextrose) at 30 °C. For the selection of transformants antibiotic resistance against G418 (200 μg/mL, Genaxxon, Ulm, Germany) was used. YPD full medium was supplemented with 10 mM methionine for the repression of *MET17*-promoter driven reporter gene expression. Minimal media (SD: 1.7 g/L yeast nitrogen base (YNB) without amino acids and NH_4_SO_4_, 20 g/L glucose, 2 g/L asparagine; and CSM: SD + 0.69 g/L CSM complete synthetic medium and CSM-Met: SD + 0.69 g/L CSM Single Drop-Out: -Met) were used for the induction of the *MET17* promoter. Regulation of gene expression controlled via *SsMET17p* was tested in the presence or absence of 2mM methionine or cysteine in CSM-Met medium. Solid media were prepared by the addition of 2% agar prior to autoclaving. *Escherichia coli* DH5α was used as host for plasmids and propagated in 2xYT (1.6% bacto peptone, 1% yeast extract, 0.5% NaCl) with 100 μg/mL ampicillin at 37 °C.

### Plasmid constructs

Plasmids pE065 (pRS417-*SsTEF*p*-lacZ-SAK1*) and pE074 (pRS417-*SsMET17*p-*lacZ*-*SAK1*) were described previously (Kayacan et al. 2019). Plasmid pE196 is a pUC57 based plasmid carrying a synthetic *S. schoenii* Histone *H4*-*GFP* fusion gene (Genscript, Piscataway NJ, USA). Plasmid pE284 (pRS417-*SsMET17*p-Ss*H4*-*GFP*-*SAK1*) was generated by cleaving pE074 with *Eco*RI, which removes most of the *lacZ* gene and inserting the *SsH4-GFP* portion from pE196 via in vivo recombination in *S. cerevisiae* (Wendland 2003). To this end the Ss*H4*-*GFP*-fragment was amplified from pE196 with primers #1140 (5’- gtgtaattatatcattttaccttcttttttactatactcgagtttATGTCCGGTCGTGGTAAAGG-3’); lower case letters indicate the homology region to the 3’-end of the *MET17*-promoter, upper case letters indicate the 5’-end of the *S. schoenii* Histone *H4*-gene) and #1141 (5’- tttgtgcaattttctctgaggaggctagatctggcgcgccggatcTATGCGTCCATCTTTACAGTC-3’); lower case letters indicate the homology region to *SAK1*, upper case letters correspond to the end of the *ScURA3*-terminator used as terminator for the *SsH4-GFP*-constuct) and co-transformed with the linear pE074 into BY4741. Correct plasmid assembly was verified by PCR and sequencing. DNA of *S. cerevisiae* transformants was prepared and plasmids were retrieved by transformation into *E. coli* as described (Kayacan et al. 2019). Plasmid DNA was prepared using the PureYield Plasmid Midiprep System (Promega, Walldorf, Germany).

### Fungal transformation

*S. cerevisiae* was transformed using the LiAc/single-stranded carrier DNA/PEG method developed by Gietz and Woods (2002) with the addition of DMSO (Kawai et al. 2010). *S. schoenii* was transformed as described (Kaimenyi et al. 2023). *S. schoenii* integrates linear DNA preferentially at ectopic positions. Thus, plasmids E074 and E284 were cut with *Kpn*I/*Sac*I to release the *SsMET17*p-*lacZ*-*SAK1* or *SsMET17*p-*SsH4*-*GFP-SAK1* fragments from their vectors, respectively, while the *SsTEF1*p-*lacZ-SAK1* fragment was released from pE065 via *Xho*I/*BamHI*. Linear DNAs were then transformed into *S. schoenii*. The *SsTEF1*p-*lacZ* fragment was co-transformed with *YES1* PCR product amplified from pYES1 (Kayacan et al. 2019) using primers #9 (5’-gaagcttcgtacgctgcaggtc-3’) and #10 (5’-tctgatatcatcgatgaattcgag-3’). Transformants were selected based on G418 resistance and the presence of the constructs was verified by PCR and subsequent functional assays. This yielded *S. schoenii* transformants G218 (*S. schoenii* with *SsTEF1p*-*lacZ*, YES1), G427, G428 and G429 (*S. schoenii* with *SsMET17*p-*lacZ-*SAK1) and G447 and G448 (*S. schoenii* with *SsMET17*p-H4-GFP-SAK1).

### Microscopy

*Saccharomycopsis* cells were propagated as described and imaged using an Axio Imager microscope equipped with a pco.edge 4.2 camera. Images were acquired and processed using VisiView 5 software (Visitron Systems GmbH, Puchheim, Germany) and Fiji (Schindelin et al. 2012).

### Testing of β-galactosidase activity

*LacZ* expression of strains G427, G428, G429 and G218 was assayed using plate- or liquid phase ONPG (ο-nitrophenyl galactopyranoside)-assays. For plate assays, strains were grown in YPD o/n, washed once with H_2_0 and then 2.5 μL of culture (∼ 1 × 10^6^ cells/mL) were spotted on YPD + 10 mM methionine plates or 2.5 μL of culture (∼ 1 × 10^7^ cells/mL) were spotted on minimal medium plates (CSM, CSM-Met and CSM-Met supplemented with either 2 mM methionine or 2 mM cysteine). Plates were incubated at 30 °C for three days. Then 5 μl of X-gal (10 mg/mL) were spotted onto each colony and plates were incubated for up to two hours before photography. The ONPG-assay was carried out as described previously (Ravasio et al. 2014). Briefly, cells were grown o/n in either YPD + 10 mM methionine or incubated in minimal medium (SD) at 30 °C. The OD_600_ was measured and samples were prepared in technical duplicates: 2 × 100 μL cell suspensions were centrifuged, washed and resuspended in 50 μl H_2_O each. Cells were disrupted by three cycles of freezing in liquid nitrogen and thawing. To these samples 75 μL Z- buffer (60 mM Na_2_HPO_4_ × 2H_2_O, 40 mM NaH_2_PO_4_ × 2H_2_O, 10 mM KCl, 1 mM MgSO_4_ × 7H_2_O) containing 4 mg/mL ONPG was added. Samples were incubated at 37 °C for 30 min. The enzymatic reaction was stopped by adding 125 μl 1 M Na_2_CO_3_. Samples were centrifuged to remove the cell debris and 200 μl of twofold diluted supernatant were measured three times at A_405_ and A_550_ (Multiskan FC Microplate Photometer and SkanIT RE Software 6.1.1, ThermoFisher Scientific) to calculate Miller units (MU = 1000 x (OD_405_ – (1, 75 x OD_550_) / (t [min] x V [ml] x OD_600_).

## Results

### Regulatable promoters in *Saccharomycopsis schoenii*

**Fig. 1:**
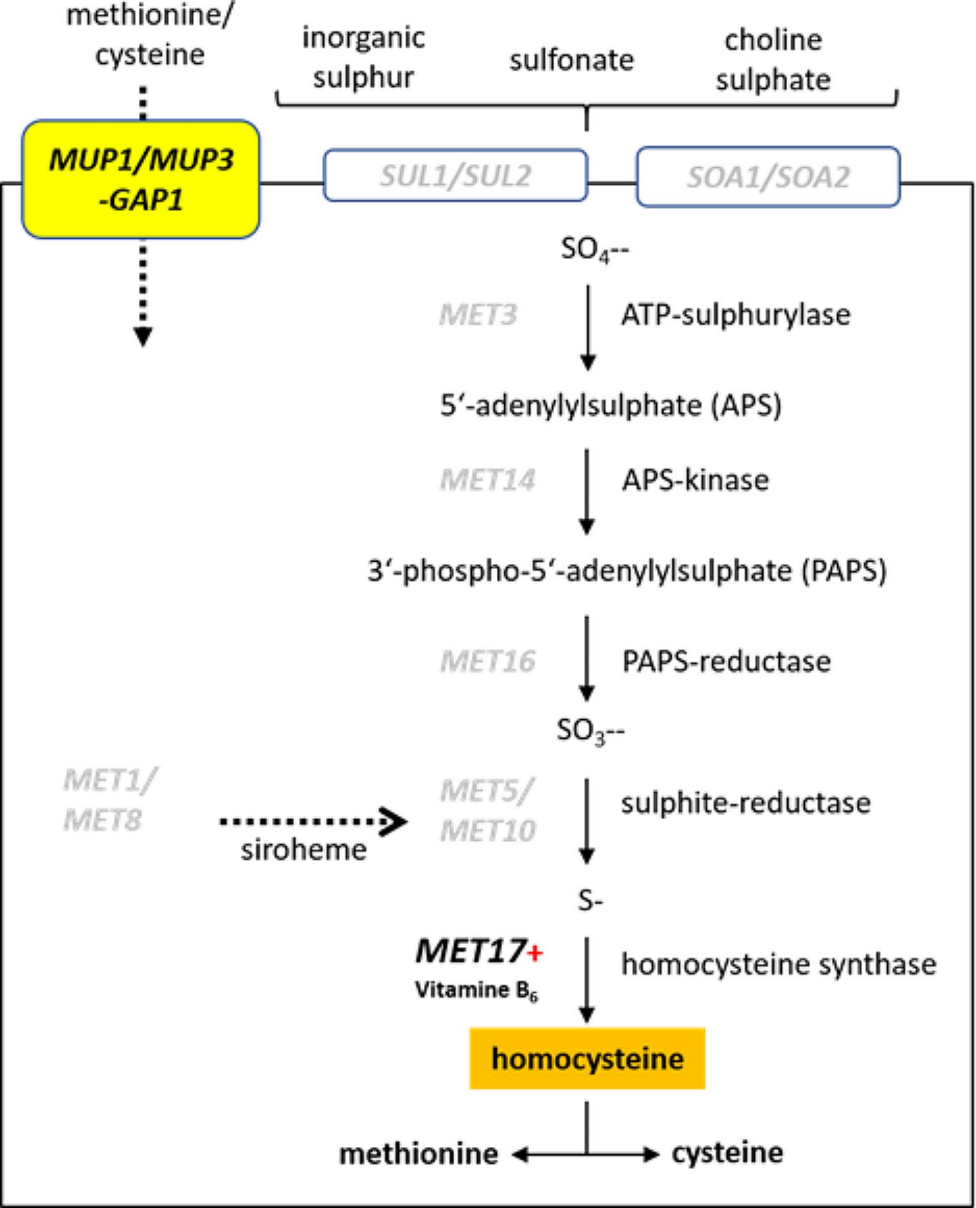
Sulphate uptake and assimilation pathway. Genes, proteins and intermediate products are shown. *MET* genes in light grey are absent in *Saccharomycopsis schoenii* and other *Saccharomycopsis* species resulting in methionine auxotrophy requiring uptake of sulphur containing amino acids via permeases encoded e.g. by *MUP1*

Comparative genomics has shown that *Saccharomycopsis* species lack 10 genes required for the uptake and assimilation of sulphate (Fig. 1). Yet, starvation for methionine still results in upregulation of sulfur metabolic processes including the genes *CYS3*, *MET2* and *MET17;* particularly, *MET17* was found to be highly upregulated under methionine deprivation (Junker et al. 2019). In *S. cerevisiae MET*-pathway genes are regulated by basic loop–helix–loop (bHLH) protein Cbf1, which recognizes the E-box consensus sequence CACGTG, and the leucine-zipper transcriptional activator Met4 (Kuras et al. 1997; Blaiseau and Thomas 1998). Homologs of these *S. cerevisiae* genes were found in the *S. schoenii* genome, suggesting that these proteins take part in the regulation of e.g. *SsMET17* during starvation. Additionally, two Cbf1 binding sites were found in the *SsMET17* promoter at -371 to -377 (CACGTG) and − 611 to -617 (CATGTG). As *MET17* is highly upregulated under methionine starvation we tested the promoter of this gene - encompassing a 1 kb upstream region - for regulated heterologous gene expression.

### MET17-promoter driven reporter gene constructs

**Fig. 2:**
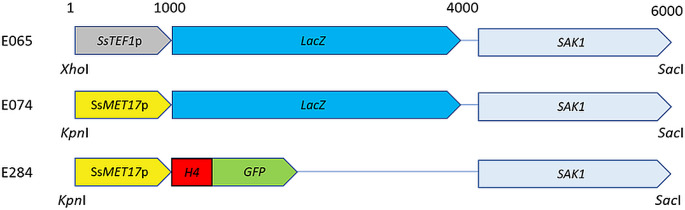
Reporter gene constructs used in this study. *lacZ* and *H4-GFP* reporter gene construct under the control of either the *S. schoenii TEF1* (plasmid E065) or *MET17* promoters (plasmids E074 and E284) were used. Plasmids were assembled in *S. cerevisiae* using in vivo recombination (for E065 and E074 see Kayacan et al. 2019). E284 was generated by exchanging the *lacZ* gene for the *H4-GFP* (see materials and methods). For transformation the cassettes were released from the vector backbone by restriction digest with the indicated enzymes. *S. schoenii* transformants were selected for G418 resistance provided by *SAK1* and *YES1*. Approximate sizes (in bp) of the constructs are shown

As reporter genes to be placed under the control of *SsMET17* we chose *lacZ* and *GFP*. To localize the green fluorescence protein in *S. schoenii* we employed a histone *H4-GFP* fusion construct (Fig. 2). Since linear DNA is preferentially integrated at ectopic loci the integration of the cassettes was untargeted. Transformants were selected based on their resistance to G418 provided by the selection markers. Integrity of the cassette after integration was verified by PCR (not shown). This yielded the transformants G218 with the *SsTEF1p-lacZ* construct to register expression of *lacZ* via a strong constitutive promoter, G427/G428/G429 carrying the *SsMET17p-lacZ* construct and G447/G448 with the *SsMET17*p-*H4-GFP* construct.

### Plate assays revealing methionine-regulatable β-galactosidase expression

**Fig. 3:**
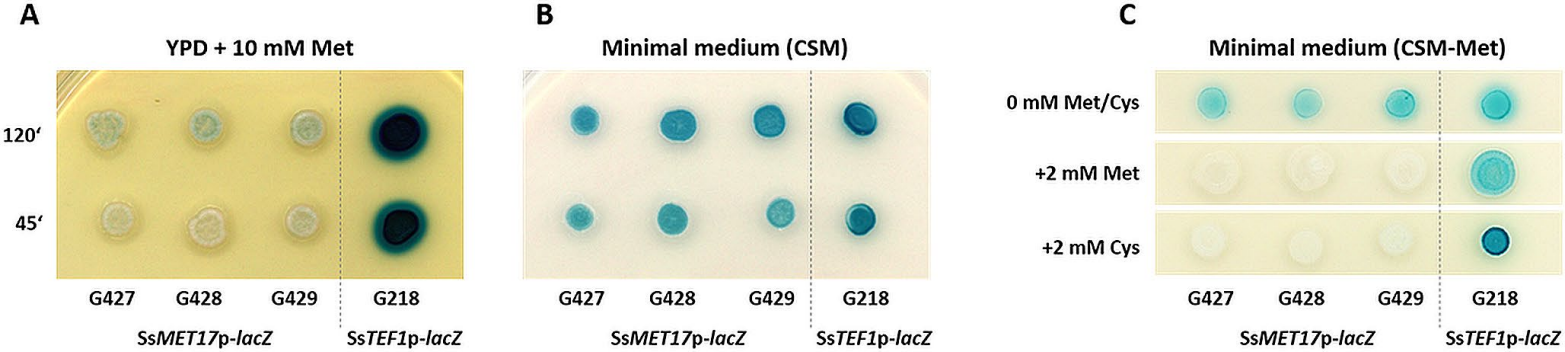
Colony level analysis of *SsMET17*-regulated *lacZ* gene expression in *S. schoenii*. Cells of strains with the indicated reporter gene constructs were spotted either on YPD + methionine plates and on minimal media CSM-plates and incubated for three days at 30 °C. To visualize β-galactosidase activity 5 μL of an X-gal solution (10 mg/mL) was spotted on each colony. Methionine was sufficient to down regulate *SsMET17*-dependent LacZ activity (**A**), while starvation induced expression of *lacZ* via the *SsMET17* promoter (**B**). Addition of methionine or cysteine to minimal medium blocked *lacZ* expression (**C**). *SsTEF1*-driven *lacZ* expression in contrast was constitutive

To analyze the regulation of heterologous gene expression via the *SsMET17*-promoter we spotted transformants on either YPD medium supplemented with additional methionine or on CSM minimal medium plates and incubated them for three days at 30 °C. Then X-gal was applied on the colonies to monitor β-galactosidase activity (Fig. 3): The *SsMET17* promoter was shut down effectively on medium containing 10 mM methionine, although we reported some leakiness after a prolonged incubation (120 min, Fig. 3A). On the other hand, expression of the reporter by the *SsMET17* promoter was strongly induced on minimal CSM medium (Fig. 3B). To investigate if the *SsMET17*-promoter can be shut off by organic sulphur compounds we added either 2 mM methionine or cysteine to the solid medium (Fig. 3C). While the control spots showed LacZ-expression, addition of methionine/cysteine blocked its expression. The expression of β-galactosidase via the *SsTEF1* promoter was very strong and constitutive under both conditions as expected. This indicates that the *SsMET17* promoter is a useful tool for regulated gene expression in *S. schoenii*.

### Liquid phase analysis of methionine-regulatable β-galactosidase expression

**Fig. 4:**
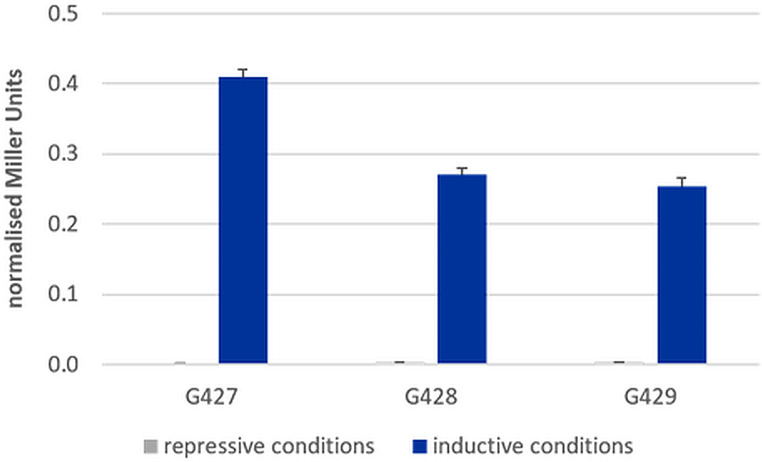
SsMET17-promoter activity in liquid-phase β-galactosidase assays. The different *Saccharomycopsis* strains shown were processed as described in *Materials and methods*. The β-galactosidase activities were calculated as Miller units shown as relative values with respect to constitutive *lacZ* expression via the *SsTEF1* promoter. Error bars represent relative standard deviations of experiments performed in technical duplicates

We used a liquid-phase ONPG assay to quantify the *SsMET17*-promoter-regulated β-galactosidase activity in either induced (minimal medium) or repressed (YPD) conditions (Fig. 4). The average absolute Miller Units ranged between 7.7 and 10.7 under repressed (YPD) and 396.2 to 639.7 under induced conditions (SD) for Ss*MET17*p-*LacZ* mutants and reached 3087.4 (YPD) and 1560.5 (SD) for the *SsTEF*p-*LacZ* mutant. Normalization of average Miller Units (relative to *SsTEF*p-*LacZ*) allowed comparison of *SsMET17*-driven β-galactosidase activity under repressive and inductive conditions: Under inducing conditions in minimal medium the *SsMET17*-driven β-galactosidase activity reached ∼ 41% of that obtained via expression of *lacZ* from the *SsTEF1*-promoter, while this relative activity was ∼ 0.3% under repressing conditions. The normalized β-galactosidase activity in minimal medium compared to YPD was increased on average by > 100-fold. (Fig. 4).

### Histone *H4-GFP* expression regulated by the *S. schoenii MET17*-promoter

**Fig. 5:**
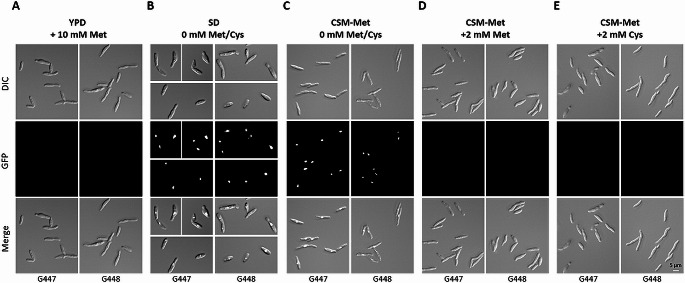
Regulation of *SsMET17*-dependent *H4*-*GFP* reporter gene activity. Cells of two independent transformants were incubated overnight (for 22 h) under repressive conditions (**A**) or inducing conditions (**B**, **C**). Addition of methionine or cysteine blocks expression of the GFP-reporter even under inducing conditions (**D**, **E**). Representative images of cells are shown. Brightfield images (DIC, upper part) and images showing GFP-fluorescence (GFP, center) were merged in an overlay (Merge, lower part). Size bar is 5 μm

We also analyzed heterologous gene expression in vivo using a *SsMET17*-driven histone *H4-GFP* reporter gene. We chose an *H4-GFP* fusion construct instead of *SsMET17*-promoter driven *GFP* expression to be able to localize the GFP-signal to the nucleus and avoid that low-level expression would be scattered in the cytoplasm. Cells grown o/n in YPD + 10 mM methionine showed no *GFP*-signal indicating that expression of the reporter gene was practically shut off (Fig. 5A). Conversely, o/n incubation in minimal SD medium strongly induced the reporter gene and resulted in bright nuclear *GFP*-fluorescence (Fig. 5B). Similar results were obtained with CSM-methionine drop out medium (CSM-Met). Addition of 2 mM methionine or 2 mM cysteine to CSM-Met efficiently down-regulated H4-GFP expression providing a range of media to control *SsMET17*-promoter activity (Fig. 5C-E).

**Fig. 6:**
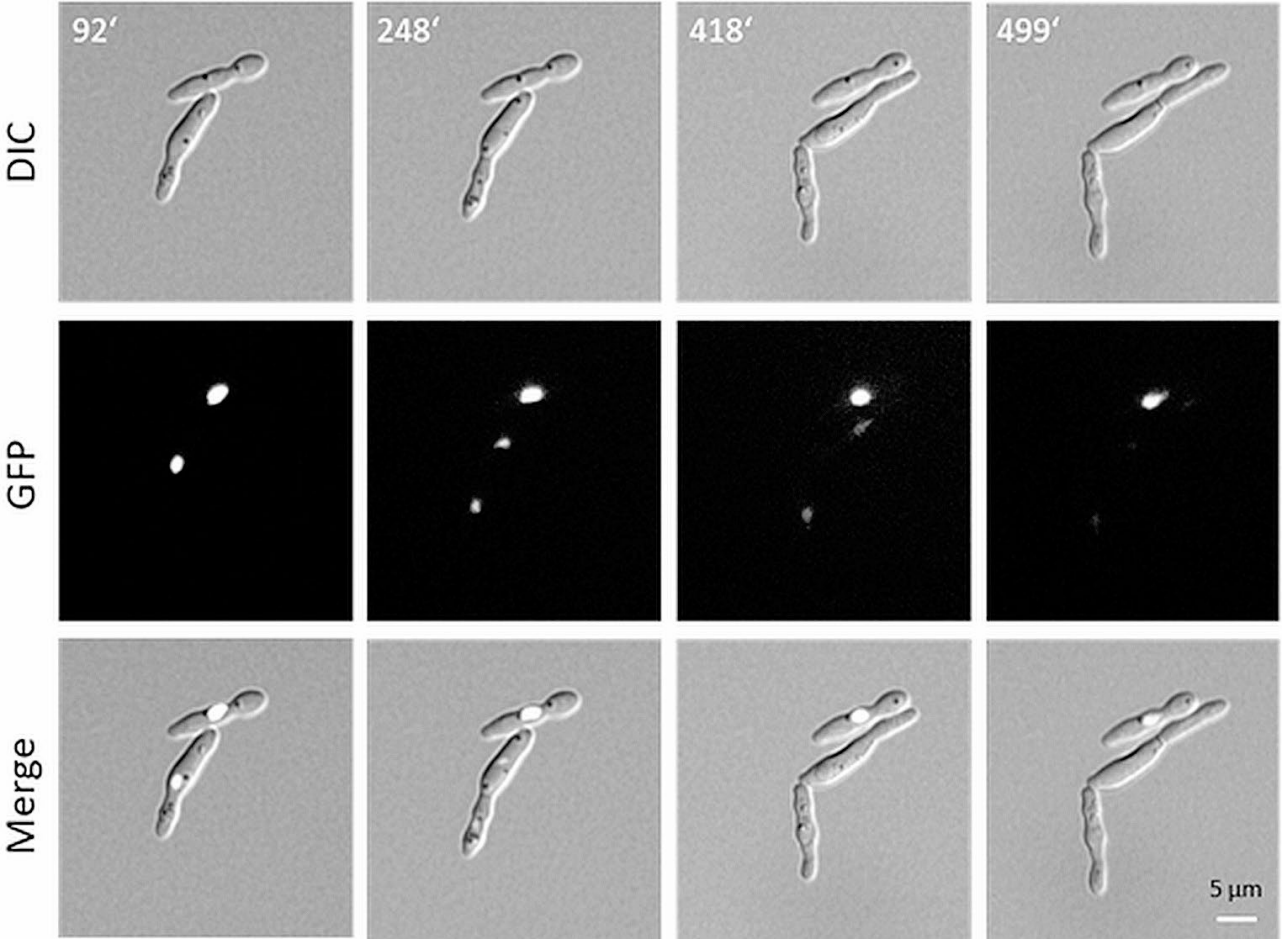
Time-lapse analysis of *H4-GFP* localization. A sequence of four time points is shown with *S. schoenii* cells of strain G447 (*SsMET17p-H4-GFP*) that were transferred from *MET*-promoter inducing to repressive conditions. At the indicated time points (in min) Brightfield (top row) and fluorescent images (middle row) were acquired and merged (bottom row). Size bar is 5 μm

To monitor the time required to lose the nuclear GFP signal we used time-lapse microscopy. Cells induced o/n in minimal medium were placed on deep-well microscopy slides containing YPD + methionine solidified with agarose. Then GFP-fluorescence was monitored over time. Resting cells maintained their nuclear GFP signal even after more than eight hours suggesting that fluorescence is not diminished by influx of untagged histone H4 (upper cell in Fig. 6). Dividing cells, however, lost their *GFP*-signal after two cell generations. Signal intensity in the dividing cell at timepoint 92’ compared to 248’ indicates that the amount of *H4*-*GFP* is distributed equally between mother and daughter nuclei suggesting that *SsMET17*-driven *H4-GFP* production is rapidly turned off after the switch to methionine-containing medium and wild type untagged histone H4 is incorporated into replicated DNA. (lower cells in Fig. 6).

## Discussion

Regulatable promoters are very useful tools to either up-regulate or down-regulate the expression of genes, particularly of essential genes or genes required for specific processes (Delic et al. 2013). A number of promoters have been established in various systems including sugar-, amino-acid and vitamin regulatable promoters. Often this requires metabolic changes to regulate these promoters, which places some constraints on the conditions under which cells can then be studied (Fuller et al. 2018). However, metabolic promoters are often quite robust in that they provide a means to shut-down promoter activity (almost) completely, e.g. in the case of *MAL*- and *GAL*-promoters for *S. cerevisiae* (Mumberg et al. 1994; Meurer et al. 2017). Other regulatable promoters that do not require changes in the medium composition are *tet*-regulatable or light-regulatable promoter systems (Wishart et al. 2005; Hurley et al. 2012).

Previously, a set of constitutive *Saccharomycopsis* promoters was identified via reporter gene analyses in *S. cerevisiae* (Kayacan et al. 2019). This study now advances the repertoire for gene-function analyses in *S. schoenii* with the characterization of the *SsMET17* promoter. In other yeasts, use of the regulatable *MET3* promoter has been described (Cherest et al. 1985; Care et al. 1999; Dünkler and Wendland, 2007). Since the *MET3* gene is not present in *Saccharomycopsis*, we picked the *SsMET17* promoter based on available RNAseq expression data (Junker et al. 2019).

Our data corroborate the RNAseq results since under inducing conditions *SsMET17* expression was found to be very strong. Importantly, expression can be turned off conveniently by an overnight incubation in methionine containing medium. This allows liquid-phase or microscopic studies. Plate assays, however, revealed that after prolonged incubation under restrictive conditions some leakiness occurs (see Fig. 3A). This may be due to colony dynamics in that cells in the center of a colony where cells may relatively quickly experience starvation conditions and thus induce *MET*-promoter activity (Cap et al. 2015).

The regulation of *MET*-promoters in *S. cerevisiae* requires protein complexes with Cbf1, the centromere binding factor 1. Cbf1 binds as a homodimer to CACGTG consensus sequences present in *S. cerevisiae MET* gene promoters and the centromere DNA element *CDEI* (Cai and Davies, 1990). *CBF1* is conserved in yeasts and Cbf1-binding sites were also identified in the *SsMET17* promoter as well as in the promoters of other sulphur metabolism genes, e.g. *SsCYS3* and *SsMET2*.

The ability to induce *Saccharomycopsis* genes under starvation conditions provides an important addition to the molecular toolbox for this genus. It will be useful to discern specific functions of genes during starvation, especially since under these conditions *Saccharomycopsis* cells trigger a predacious behavior that results in penetration peg formation and the attack and subsequent killing of prey cells (Lachance et al., 1997; Junker et al. 2018, 2019). The *SsMET17* promoter allows the upregulation of genes during starvation, i.e. at the onset of predacious behavior.

## Data Availability

No datasets were generated or analysed during the current study.
